# Multicenter Evaluation of AI-generated DIR and PSIR for Cortical and Juxtacortical Multiple Sclerosis Lesion Detection

**DOI:** 10.1148/radiol.221425

**Published:** 2023-02-07

**Authors:** Piet M. Bouman, Samantha Noteboom, Fernando A. Nobrega Santos, Erin S. Beck, Gregory Bliault, Marco Castellaro, Massimiliano Calabrese, Declan T. Chard, Paul Eichinger, Massimo Filippi, Matilde Inglese, Caterina Lapucci, Andrzej Marciniak, Bastiaan Moraal, Alfredo Morales Pinzon, Mark Mühlau, Paolo Preziosa, Daniel S. Reich, Maria A. Rocca, Menno M. Schoonheim, Jos W. R. Twisk, Benedict Wiestler, Laura E. Jonkman, Charles R. G. Guttmann, Jeroen J. G. Geurts, Martijn D. Steenwijk

**Affiliations:** From the MS Center Amsterdam, Anatomy & Neurosciences, Amsterdam Neuroscience, Amsterdam UMC, Vrije Universiteit Amsterdam, De Boelelaan 1117, Amsterdam, the Netherlands (P.M.B., S.N., F.A.N.S., M.M.S., J.J.G.G., M.D.S.); Translational Neuroradiology Section, National Institute of Neurological Disorders and Stroke, National Institutes of Health, Bethesda, Md (E.S.B., D.S.R.); Department of Neurology, Icahn School of Medicine at Mount Sinai, New York, NY (E.S.B.); Bio-imaging Institute, University of Bordeaux, Bordeaux, France (G.B.); Neurology Section, Department of Neuroscience, Biomedicine and Movement Sciences, University of Verona, Verona, Italy (M. Castellaro, M. Calabrese); Department of Information Engineering, University of Padova, Padova, Italy (M. Castellaro); NMR Research Unit, Queen Square MS Centre, Department of Neuroinflammation, UCL Queen Square Institute of Neurology, Faculty of Brain Sciences, University College London, London, UK (D.T.C.); National Institute for Health Research University College London Hospitals Biomedical Research Centre, London, UK (D.T.C.); Departments of Neuroradiology (P.E., B.W.) and Neurology (M.M.), School of Medicine, Klinikum Rechts der Isar, Technical University of Munich, Munich, Germany; Neuroimaging Research Unit, Division of Neuroscience Neurology Unit, IRCCS San Raffaele Scientific Institute Vita-Salute San Raffaele University, Milan, Italy (M.F., P.P., M.A.R.); Department of Neuroscience, Rehabilitation, Ophthalmology, Genetics, Maternal and Child Health, University of Genova, Genoa, Italy (M.I., C.L.); IRCCS Ospedale Policlinico San Martino, Largo Rosanna Benzi, Genoa, Italy (M.I., C.L.); Center for Neurologic Imaging, Department of Radiology, Brigham and Women’s Hospital, Harvard Medical School, Boston, Mass (A.M., A.M.P., C.R.G.G.); Department of Radiology and Nuclear Medicine, MS Center Amsterdam, Amsterdam Neurosciences, Amsterdam UMC, Vrije Universiteit Amsterdam, Amsterdam, the Netherlands (B.M.); Department of Epidemiology and Data Science, Amsterdam University Medical Center, Amsterdam, the Netherlands (J.W.R.T.); Anatomy & Neurosciences, Amsterdam UMC, Vrije Universiteit Amsterdam, Amsterdam, the Netherlands (L.E.J.); and Amsterdam Neuroscience, Brain Imaging and Neurodegeneration, Amsterdam, the Netherlands (L.E.J.).

## Abstract

**Background:**

Cortical multiple sclerosis lesions are clinically relevant but inconspicuous at conventional clinical MRI. Double inversion recovery (DIR) and phase-sensitive inversion recovery (PSIR) are more sensitive but often unavailable. In the past 2 years, artificial intelligence (AI) was used to generate DIR and PSIR from standard clinical sequences (eg, T1-weighted, T2-weighted, and fluid-attenuated inversion-recovery sequences), but multicenter validation is crucial for further implementation.

**Purpose:**

To evaluate cortical and juxtacortical multiple sclerosis lesion detection for diagnostic and disease monitoring purposes on AI-generated DIR and PSIR images compared with MRI-acquired DIR and PSIR images in a multicenter setting.

**Materials and Methods:**

Generative adversarial networks were used to generate AI-based DIR (*n* = 50) and PSIR (*n* = 43) images. The number of detected lesions between AI-generated images and MRI-acquired (reference) images was compared by randomized blinded scoring by seven readers (all with >10 years of experience in lesion assessment). Reliability was expressed as the intraclass correlation coefficient (ICC). Differences in lesion subtype were determined using Wilcoxon signed-rank tests.

**Results:**

MRI scans of 202 patients with multiple sclerosis (mean age, 46 years ± 11 [SD]; 127 women) were retrospectively collected from seven centers (February 2020 to January 2021). In total, 1154 lesions were detected on AI-generated DIR images versus 855 on MRI-acquired DIR images (mean difference per reader, 35.0% ± 22.8; *P* < .001). On AI-generated PSIR images, 803 lesions were detected versus 814 on MRI-acquired PSIR images (98.9% ± 19.4; *P* = .87). Reliability was good for both DIR (ICC, 0.81) and PSIR (ICC, 0.75) across centers. Regionally, more juxtacortical lesions were detected on AI-generated DIR images than on MRI-acquired DIR images (495 [42.9%] vs 338 [39.5%]; *P* < .001). On AI-generated PSIR images, fewer juxtacortical lesions were detected than on MRI-acquired PSIR images (232 [28.9%] vs 282 [34.6%]; *P* = .02).

**Conclusion:**

Artificial intelligence–generated double inversion-recovery and phase-sensitive inversion-recovery images performed well compared with their MRI-acquired counterparts and can be considered reliable in a multicenter setting, with good between-reader and between-center interpretative agreement.

Published under a CC BY 4.0 license.

*Supplemental material is available for this article.*

See also the editorial by Zivadinov and Dwyer in this issue.

## Graphical abstract

**Figure:**
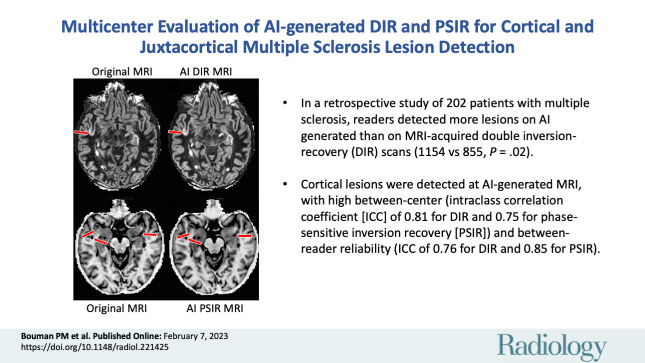

**Summary**

Artificial double inversion-recovery and phase-sensitive inversion-recovery MRI scans were generated from multicenter input data, with high between-center and between-reader reliability for detection of cortical and juxtacortical multiple sclerosis lesions.

**Key Results**

■ In a retrospective study of 202 patients with multiple sclerosis, readers could detect more lesions on artificially generated than on MRI-acquired double inversion-recovery (DIR) images (1154 vs 855; *P* = .02).
■ Cortical lesions could be detected at artificial MRI, with high between-center (intraclass correlation coefficient [ICC] of 0.81 for DIR and 0.75 for phase-sensitive inversion recovery [PSIR]) and between-reader reliability (ICC of 0.76 for DIR and 0.85 for PSIR).

## Introduction

Multiple sclerosis is an inflammatory, demyelinating, and neurodegenerative disease of the central nervous system that leads to a variety of physical and cognitive disabilities (1–3). Although multiple sclerosis lesions can affect the entire central nervous system, they are most conspicuous in the white matter at MRI (4–6). However, immunohistochemical staining revealed that multiple sclerosis lesions also frequently occur in the cortical gray matter (7). Cortical gray matter lesions are known to be related to disability and cognition and could thus play an important role in monitoring disease progression in patients with multiple sclerosis (8–10). Cortical gray matter lesions visible at MRI were found to be a pathologic hallmark for multiple sclerosis, which has led to expansion of the diagnostic criteria from only juxtacortical lesions (ie, touching the cortex) to lesions that are juxtacortical *or* cortical (11).

Due to their small size and low contrast relative to normal-appearing gray matter, cortical lesions are inconspicuous on images acquired with conventional clinical MRI sequences (eg, T1-weighted, T2-weighted, and fluid-attenuated inversion-recovery, or FLAIR, sequences) (12). Improved visualization of cortical lesions has been achieved with the development of advanced MRI techniques, such as double inversion-recovery (DIR) and phase-sensitive inversion-recovery (PSIR) sequences (4,5,7,13,14). However, these sequences are largely absent in routine diagnostic and clinical trial evaluation protocols due to their substantial acquisition times (ie, 10–15 minutes). Pilot studies using artificial intelligence (AI) have recently enabled the generation of artificial DIR images from routine clinical MRI protocols (ie, combinations of T1- and proton density–/T2-weighted sequences) (15,16). Histopathologic validation of AI-generated DIR images vis-à-vis MRI-acquired images showed equal sensitivity and specificity values (17). To leverage the potential of AI-generated DIR and PSIR images to serve as an alternative to MRI-acquired DIR and PSIR images, multicenter data evaluation is crucial for understanding their value in a clinical or research setting with hardware and sequence parametrization variability.

The primary aim of our study was to evaluate cortical and juxtacortical multiple sclerosis lesion detection for diagnostic and disease monitoring purposes on AI-generated DIR and PSIR images compared with MRI-acquired DIR and PSIR images in a multicenter setting.

## Materials and Methods

### Study Design and Participants

Data for our study were retrospectively collected between February 2020 and January 2021 from seven academic medical centers (National Institutes of Health, United States; University College London, United Kingdom; Technical University of Munich, Germany; University of Genoa, Italy; IRCCS San Raffaele Scientific Institute, Italy; Università di Verona, Italy; and Amsterdam UMC, the Netherlands). Some of the data were previously analyzed for different purposes (Appendix S1). All patients provided written informed consent before study commencement and data sharing. Studies were approved by the institutional ethics review boards of all participating centers.

### Collection of MRI Data

Each participating center was requested to provide whole-brain MRI data of at least 20 patients diagnosed with multiple sclerosis according to McDonald or Poser criteria (11,18,19). Inclusion and exclusion criteria were presence or absence of MRI scans acquired at clinical field strength of 1.5 T or 3.0 T, including a DIR and/or PSIR sequence (ideally three-dimensional, near 1-mm isotropic resolution), a three-dimensional T1-weighted sequence (near 1-mm isotropic resolution), and either a proton density–/T2-weighted sequence, fluid-attenuated inversion-recovery sequence, or both (3-mm sections or higher resolution). Imaging parameters are displayed in Appendix S2 (Tables S1–S5).

### Image Preprocessing and Generation of Artificial Images

An extensive description of the full image processing pipeline is provided elsewhere (15). In brief, all available contrasts of each patient were coregistered with each other with use of rigid body transformation using FLIRT (liner image registration tool, part of the Functional MRI of the Brain Software Library, or FSL, version 5.0.4; *http://fsl.fmrib.ox.ac.uk*). The resulting transformation matrixes were used to rigidly transform the data of all individuals into 1-mm Montreal Neurological Institute system standard space (spline interpolation). The skull was removed from the data by using bet-premask (FSL). Variance scaling, but not any other intensity or bias field correction, was applied to each individual contrast in each patient.

Next, two separate generative adversarial networks (U-Net–like convolutional networks) were trained to generate either artificial DIR or PSIR images from the available clinical MRI sequences (ie, T1-weighted and proton density–/T2-weighted or fluid-attenuated inversion-recovery sequences). Images from the different centers were equally distributed into training and test sets (2:1 ratio), after which the generative adversarial networks were applied to the training sets. The code that was used to generate the artificial images is deposited at *https://github.com/MrtnStnwk/DeepContrast*.

### Lesion Identification

AI-generated DIR and PSIR images, along with their MRI-acquired counterparts, were randomly distributed for lesion assessment among seven readers, all from different centers. Each reader received 30 data sets: eight AI-generated DIR images, seven AI-generated PSIR images, and the corresponding MRI-acquired images. One randomly selected DIR and one randomly selected PSIR data set were provided to all readers for reliability calculations. The other data sets were reviewed by only one reader. Images were provided in random order and randomly left-right flipped to reduce recognition probability. Lesion detection was performed by seven authors (B.M., C.L., D.T.C., E.S.B., M.M., P.E., and P.P., all with >10 years of experience in cortical lesion detection) in accordance with consensus recommendations by the Magnetic Resonance Imaging in MS, or MAGNIMS, group for MRI-acquired as well as AI-generated DIR images and sequence-specific guidelines for MRI-acquired and AI-generated PSIR images (14,20). Readers were also asked to indicate lesion type as juxtacortical (touching but not entering cortical gray matter), leukocortical (situated in part in the cortex and in part in the adjacent white matter), intracortical (fully situated in the cortex), or infratentorial (below the cerebellar tentorium). In-depth descriptions of the procedures and lesion detection criteria are provided in Appendixes S3–S5.

### Statistical Analysis

The number of lesions detected on the AI-generated images was compared with that on the corresponding MRI-acquired images, using the latter as reference. Distribution of the data was assessed for normality with use of Kolmogorov-Smirnov tests and log-transformed if not normally distributed.

Statistical analyses were performed by three authors (P.M.B., L.E.J., and J.W.R.T., with 4, 8, and >15 years of experience, respectively). Differences in the number of detected lesions per patient were assessed using pairwise *t* tests, and differences in the number of detected lesions at the lesion subtype level were assessed using pairwise Wilcoxon signed-rank tests. Between-reader agreement (reliability; identical patient) was assessed by calculating the intraclass correlation coefficient (ICC) (two-way mixed model with absolute agreement). Calculations were based on the number of lesions identified per 20 sections (ie, 0–20; 21–40; 41–60; 60–80). Using a similar model, reliability between AI-generated and MRI-acquired DIR and PSIR images across all centers (nonidentical patients) was determined.

Post hoc precision of the AI-generated images was estimated using data from five randomly selected patients per contrast. Lesions detected on AI-generated and MRI-acquired images were matched to obtain true- and false-positive results (taking MRI-acquired images as the reference). This allowed the calculation of precision (precision = true-positive results divided by total positive results; performed by P.M.B.). Additionally, image contrast ratios were calculated for a random subsample of 14 patients to explore the origin of differences. A detailed description of these calculations is provided in Appendix S6. *P* < .05 was considered indicative of statistically significant difference.

Analyses were performed using Statistical Package for Social Sciences, version 28.0 (IBM).

## Results

### Patient Characteristics

**Figure 1:**
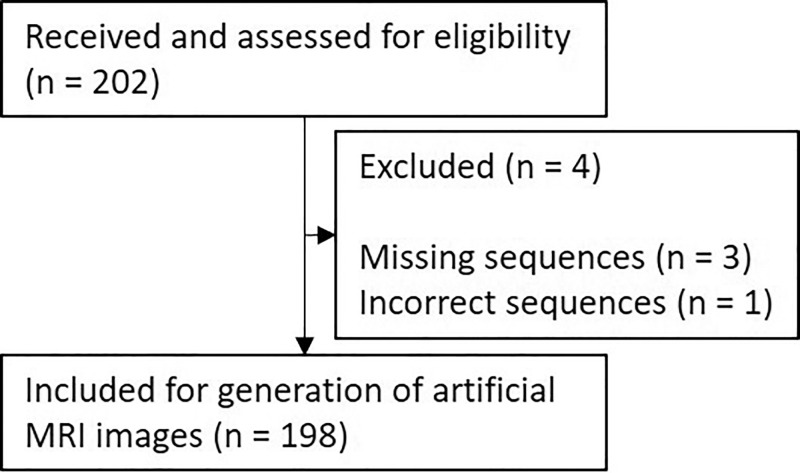
Flowchart of included and excluded patients displays number of patients from whom images were received and reasons for exclusion.

DIR images (*n* = 160) and PSIR images (*n* = 125) from a total of 202 patients with multiple sclerosis (mean age, 46 years ± 11 [SD]; 127 women) were received. Data from four patients were excluded due to missing or incorrect sequences. The training and test sets consisted of 106 and 50 patients, respectively, for DIR and 82 and 43 patients, respectively, for PSIR sequences. A flowchart of the procedure is displayed in Figure 1. The demographic characteristics of the patients for the DIR and PSIR training and test sets are displayed in Table 1.

**Table 1:**
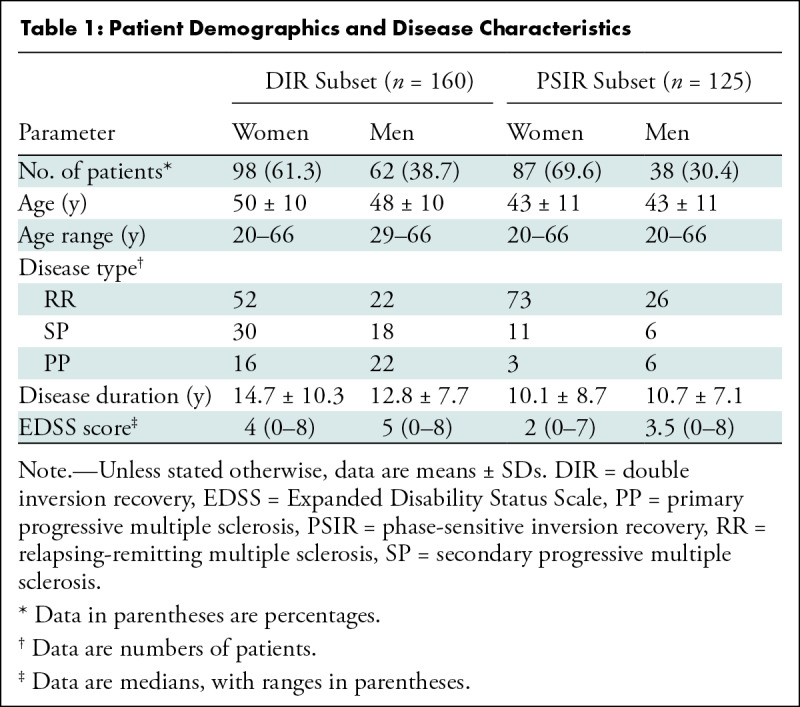
Patient Demographics and Disease Characteristics

### Lesion Detection on AI-generated Images

**Figure 2:**
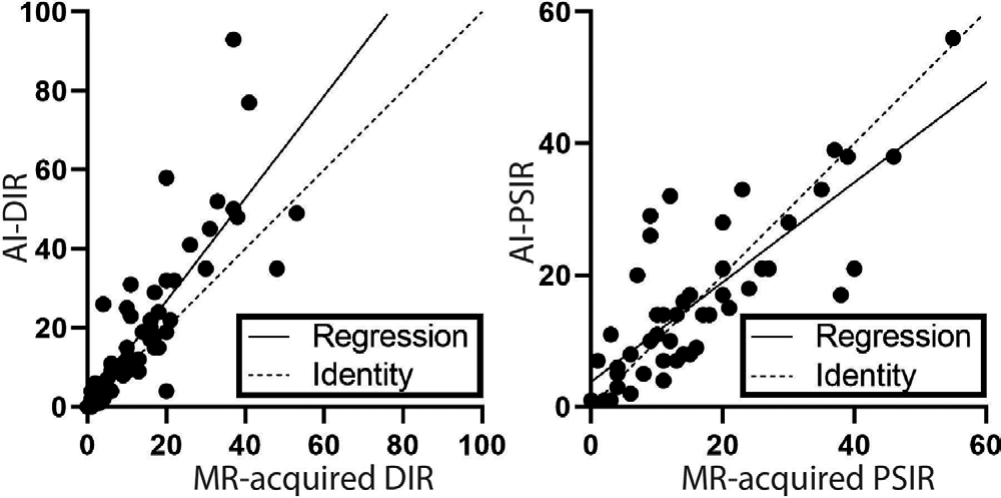
Graphical overview of detected cortical and juxtacortical lesions on artificial intelligence (AI)–generated versus MRI-acquired images. Line graphs show AI-generated double inversion-recovery (DIR) images vis-à-vis MRI-acquired DIR images per patient (left) and AI-generated phase-sensitive inversion-recovery images (PSIR) vis-à-vis MRI-acquired PSIR images (right). The solid line indicates the regression, and the dashed line indicates identity.

An overview of the number of lesions that were detected on AI-generated versus MRI-acquired DIR and PSIR images is provided in Figure 2.

**Figure 3:**
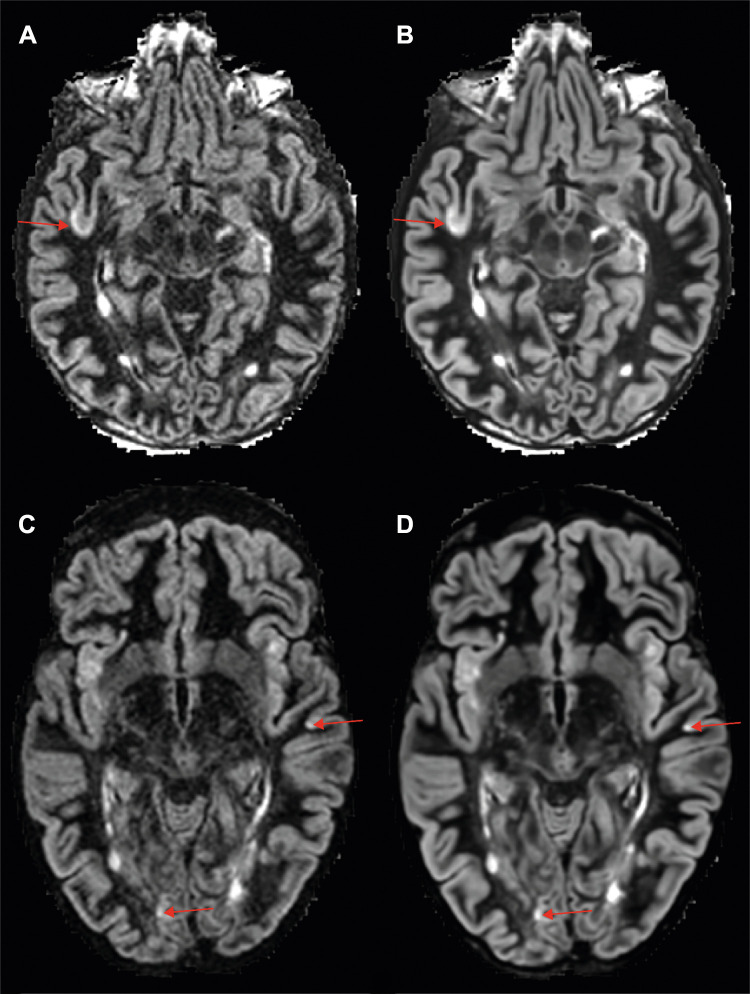
Examples of detected cortical lesions on axial double inversion-recovery (DIR) images. **(A, C)** MRI-acquired (noncontrast) and **(B, D)** artificial intelligence–generated DIR images. The arrows indicate detected intracortical lesions.

**Figure 4:**
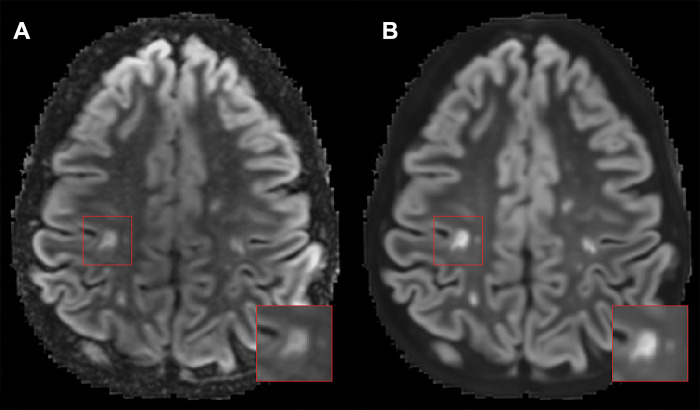
Example of a false-positive annotated lesion. **(A)** Axial MRI-acquired versus **(B)** artificial intelligence (AI)–generated double inversion-recovery images (noncontrast) in a 38-year-old woman. The inset indicates a lesion that was considered a juxtacortical lesion on the AI-generated image but was not identified as such on the MRI-acquired image due to the rim of white matter that is visible between the lesion and the cortex.

On AI-generated DIR images, a total of 1154 lesions were detected, compared with 855 on MRI-acquired DIR images. Some examples of cortical lesions that were detected on AI-generated and MRI-acquired DIR images are depicted in Figure 3. Averaged over patients, the readers detected 35% more lesions on AI-generated DIR images than on MRI-acquired DIR images, with differences ranging from 15% to 79% more lesions (*t* = 2.39, *P* = .02). The median number of detected lesions was 15 (IQR, 8–23) for AI-generated DIR and 13 (IQR, 6–20) for MRI-acquired DIR images. Reliability analysis between centers showed good agreement in the number of detected lesions on AI-generated and MRI-acquired DIR images across all assessed data sets (ICC, 0.81 [95% CI: 0.68, 0.89]). The precision of the AI-generated DIR images was 72.8% ± 13.1. An example of a lesion that appears juxtacortical on the AI-generated DIR image but subcortical (and thus a false-positive finding, as readers were specifically instructed not to mark subcortical lesions) on the MRI-acquired DIR image is presented in Figure 4.

**Figure 5:**
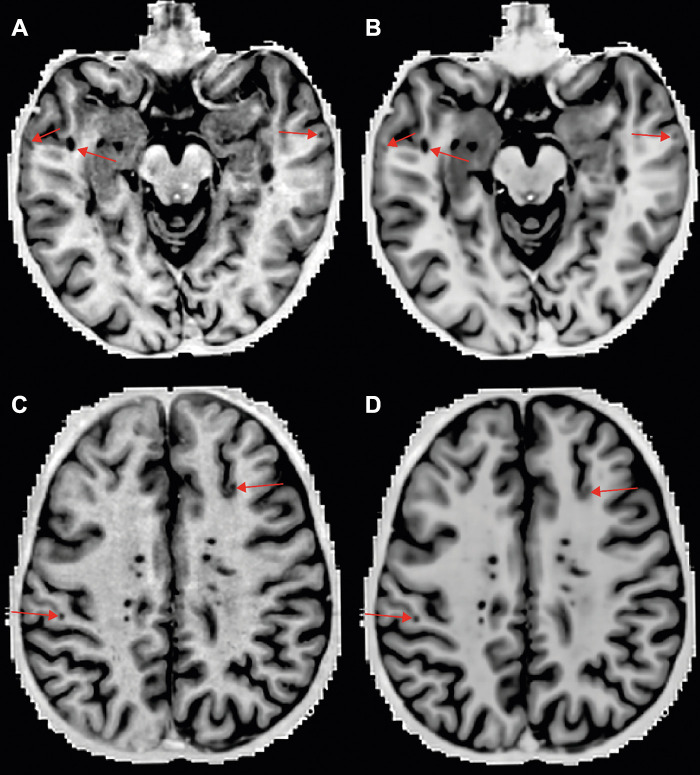
Overview of detected cortical lesions on axial phase-sensitive inversion-recovery (PSIR) images. **(A, C)** MRI-acquired versus **(B, D)** artificial intelligence–generated PSIR images. The arrows indicate detected intracortical and leukocortical lesions.

For PSIR, a total of 803 lesions were detected on AI-generated images and 814 on MRI-acquired images. Examples of cortical lesions that were detected on AI-generated and MRI-acquired PSIR images are depicted in Figure 5. The overall number of lesions detected on AI-generated PSIR images compared with MRI-acquired PSIR images was 98.9% ± 19.4, ranging from 71.6% to 116.8% for the different readers (*t = *−0.16, *P* = .87). The median number of detected lesions was 14 (IQR, 7–21) for AI-generated PSIR images and 13 (IQR, 7–22) for MRI-acquired PSIR images. Reliability analysis between centers for AI-generated PSIR images versus MRI-acquired PSIR images showed good agreement for the number of detected lesions across all assessed data sets (ICC, 0.75 [95% CI: 0.59, 0.85]). The precision of the AI-generated PSIR images was 69.5% ± 13.1.

Assessment of between-reader variability (same data set for all readers) showed good agreement for AI-generated DIR images (ICC, 0.76 [95% CI: 0.43, 0.98]), MRI-acquired DIR images (ICC, 0.85 [95% CI: 0.58, 0.99]), AI-generated PSIR images (ICC, 0.85 [95% CI: 0.59, 0.99]), and MRI-acquired PSIR images (ICC, 0.85 [95% CI: 0.59, 0.99]). The median number of lesions detected in the patient whose images were assessed by all readers was 15 (IQR, 11–22) for AI-generated DIR, 12 (IQR, 11–15) for MRI-acquired DIR, eight (IQR, five to eight) for AI-generated PSIR, and seven (IQR, five to nine) for MRI-acquired PSIR.

### Differences between Identified Lesion Subtypes on Different Sequences

**Figure 6:**
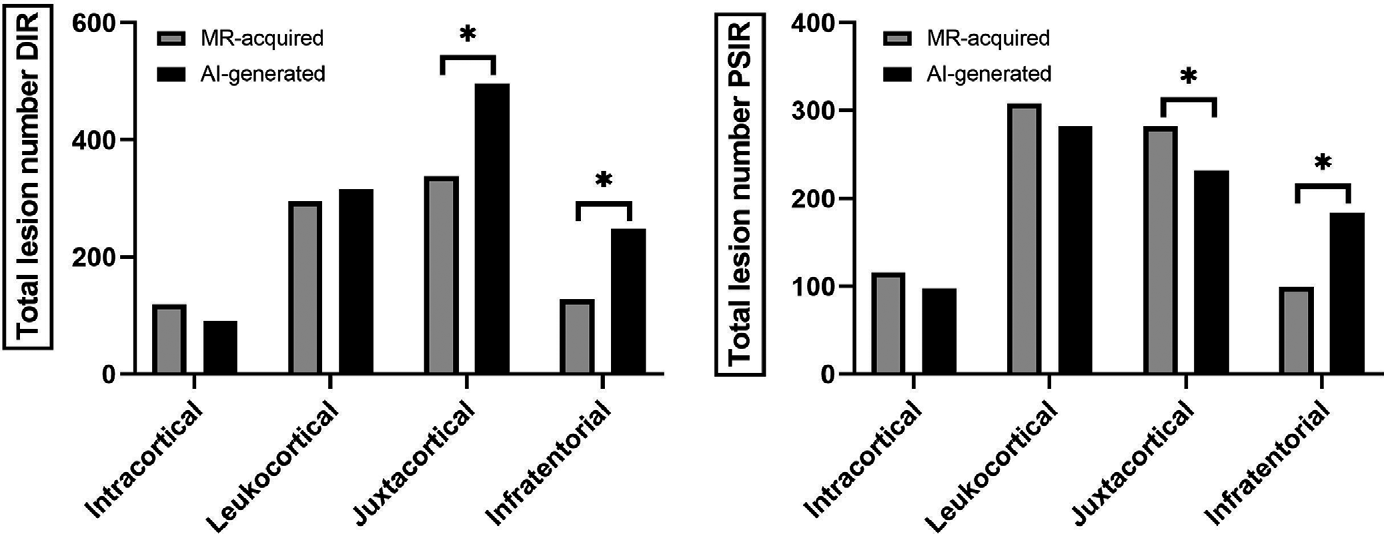
Bar graphs show the total number of detected lesions per lesion type. The left graph shows total lesion numbers that were detected on artificial intelligence (AI)–generated double inversion-recovery (DIR) images versus MRI-acquired DIR images per lesion subtype. The right graph shows an overview of total lesion numbers that were detected on AI-generated phase-sensitive inversion-recovery (PSIR) versus MRI-acquired PSIR images. * = *P* < .05.

A graphical overview of the detected differences between lesion subtypes on AI-generated versus MRI-acquired images is depicted in Figure 6. For AI-generated versus MRI-acquired DIR images, respectively, 495 of 1154 (42.9%) and 338 of 855 (39.5%) detected lesions were classified as juxtacortical (*Z* = 3.52, *P* < .001); 316 of 1154 (27.4%) and 261 of 855 (30.5%) as leukocortical (*Z* = −1.38, *P* = .17); 92 of 1154 (8.0%) and 119 of 855 (13.9%) as intracortical (*Z* = −1.52, *P* = .13); and 248 of 1154 (21.5%) and 128 of 855 (15.0%) as infratentorial (*Z* = −4.30, *P* < .001). Three of 1154 lesions (0.3%) and nine of 855 lesions (1.1%) were not classified by the reader.

For AI-generated versus MRI-acquired PSIR images, respectively, the majority of lesions were classified as leukocortical: 282 of 803 (35.1%) and 308 of 814 (37.8%) (*Z* = −0.87, *P* = .38); followed by juxtacortical lesions: 232 of 803 (28.9%) and 282 of 814 (34.6%) (*Z* = −2.42, *P* = .02); intracortical lesions: 97 of 803 (12.1%) and 116 of 814 (14.3%) (*Z* = −1.53, *P* = .13); and infratentorial lesions: 184 of 803 (22.9%) and 99 of 814 (12.2%) (*Z* = −3.37, *P* = .001). Eight of 803 lesions (1.0%) and nine of 814 lesions (1.1%) were not classified by the reader.

### Image Contrast Ratios

Contrast ratios were calculated for a random subset of 28 lesions that were detected by the readers in 14 different patients. The outcomes of the contrast ratio calculations (calculated for 28 regions) are presented in Table 2. For AI-generated versus MRI-acquired DIR images, no differences in cortical lesion versus normal-appearing gray matter contrast (0.23 ± 0.20 vs 0.26 ± 0.23, respectively; *Z* = −1.94, *P* = .053) were observed, but the contrast was higher for normal-appearing gray matter versus normal-appearing white matter in the MRI-acquired DIR images (0.43 ± 0.16 vs 0.51 ± 0.18; *Z* = −3.26, *P* = .001). For PSIR, contrast between cortical lesions versus normal-appearing gray matter was higher in MRI-acquired images (−0.08 ± 0.12 vs −0.12 ± 0.07; *Z* = −3.17, *P* = .002), and no evidence of a difference was found for the contrast ratio of normal-appearing gray matter versus normal-appearing white matter (−0.12 ± 0.08 vs −0.13 ± 0.08; *Z* = −1.07, *P* = .29).

**Table 2:**
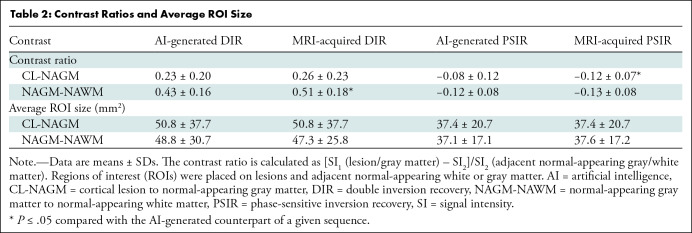
Contrast Ratios and Average ROI Size

## Discussion

Our study investigated whether otherwise inconspicuous cortical or juxtacortical multiple sclerosis lesions can be detected on artificial intelligence (AI)–generated double inversion-recovery (DIR) and phase-sensitive inversion-recovery (PSIR) images in a multicenter data set. An earlier study in a single-center setting using 1.5-T MRI showed that AI could potentially mitigate the issue of often-absent DIR sequences in clinical care and clinical trials (15). Our results demonstrate that more lesions can be detected on AI-generated DIR images than on their MRI-acquired counterparts (*t* = 2.39, *P* = .02) and an equal number of lesions on the AI-generated PSIR images as on their MRI-acquired counterparts (*t* = −0.16, *P* = .87), with good between-center (intraclass correlation coefficient [ICC], 0.81 and 0.75 for DIR and PSIR, respectively) and between-reader agreement (ICC, 0.76 and 0.85 for AI-generated and MRI-acquired DIR images, respectively, and 0.85 and 0.85 for AI-generated and MRI-acquired PSIR images). These results suggest that AI-generated DIR and PSIR images could be used in diagnostic and disease monitoring settings when MRI-acquired sequences are not available.

Our study suggests that a higher number of juxtacortical lesions can be detected on AI-generated DIR images than on MRI-acquired DIR images. This is different from earlier studies examining AI-generated DIR images, which found similar numbers of detected lesions (15,17). The higher number of detected lesions in the AI-generated DIR images was also reflected in the precision and may have emerged from the smoother appearance of the AI-generated images and the influence of AI on the cortical rim (due to white matter suppression optimization by the algorithm). This, in turn, might have influenced the differentiation between subcortical and juxtacortical lesions: Readers were specifically instructed to make this differentiation based on lesions touching (ie, juxtacortical) or not touching (ie, subcortical) the cortex. Of these, only juxtacortical lesions were to be annotated. Another explanation is the T1 component in the AI-generated images: T1-weighted images have previously been found to provide better contrast for juxtacortical lesions than T2-weighted images (21). However, the most important finding is that AI-generated DIR and PSIR images provide a tool for cortical lesion detection with which a similar number of cortical lesions can be detected compared with MRI-acquired DIR and PSIR images. These findings might contribute to diagnostic considerations for the establishment of dissemination in space and imaging protocols in multiple sclerosis care (9,11).

The results also showed high comparability between AI-generated and MRI-acquired PSIR images. Previous research found that PSIR may provide sharper contrast between gray and white matter compared with DIR and thereby enable the reader to distinguish more accurately between juxtacortical and leukocortical lesions (22). These findings seem to be reflected in our results, with more leukocortical than juxtacortical lesions detected on PSIR images. The precision of PSIR may have been reduced by false-positive findings, which were predominantly located infratentorially. This may be because T2-weighted sequences are preferred for infratentorial lesion assessment and because the AI-generated images contained a T2-weighted component. For both DIR and PSIR, the overall number of detected infratentorial lesions might have been underestimated, as three-dimensional fluid-attenuated inversion-recovery and T2-weighted spin-echo sequences are generally used for the detection of these lesions, rather than DIR or PSIR (9,23). Concordant with the previous literature, most of the detected lesions were situated in the temporal and frontal lobes (24–30). The location of the detected lesions may be relevant to unravel their role in, for example, physical or cognitive deterioration or disease progression and conversion (8,30–35). In regard to lesion detection on AI-generated PSIR images, studies are lacking in the literature. It is important, however, to note that our study does not postulate a direct comparison between AI-generated DIR and PSIR images, as different patients’ images were assessed.

This study had limitations. First, regarding in vivo data, MRI-acquired images had to be used as reference rather than histopathologic findings. Although histopathologic validation of AI-generated DIR images yielded promising results (ie, 90% specificity), histopathologic validation of AI-generated PSIR images should be addressed in future endeavors (4,17). Second, 3-mm reformatting of the images increased the difficulty of assessing small suspect lesions (eg, distinguishing between lesions and cortical vessels or Virchow-Robin spaces). However, through-plane averaging might also have improved lesion discernibility in some instances. Third, the resolution of the coronal and sagittal images was not optimized, and thus, they could not be used for lesion detection or verification. Altogether, this may have hampered cortical lesion detection to some extent. However, all these impediments were present in both AI-generated and MRI-acquired images. Future endeavors should compare lesion detection numbers on a per-protocol basis to evaluate which combination of sequences (for different vendors and two- and three-dimensional acquisitions) would generate artificial images with the highest achievable detection potential.

In conclusion, our study showed that the number of detectable cortical and juxtacortical lesions on artificial intelligence (AI)–generated double inversion-recovery (DIR) and phase-sensitive inversion-recovery (PSIR) images is at least equal to their MRI-acquired counterparts. Not only were the images generated using multicenter input data, they were also assessed for cortical and juxtacortical lesions with good between-center and between-reader agreement. This indicates that AI-generated DIR and PSIR images can serve as a practical alternative to visualize cortical pathologic abnormalities in multiple sclerosis in clinical care or (retrospective) clinical studies when their MRI-acquired counterparts are desired but absent.
